# Detection of second-line drug resistance in *Mycobacterium tuberculosis* using oligonucleotide microarrays

**DOI:** 10.1186/1471-2334-13-240

**Published:** 2013-05-24

**Authors:** Danila V Zimenkov, Olga V Antonova, Alexey V Kuz’min, Yulia D Isaeva, Ludmila Y Krylova, Sergey A Popov, Alexander S Zasedatelev, Vladimir M Mikhailovich, Dmitry A Gryadunov

**Affiliations:** 1 Engelhardt Institute of Molecular Biology, Russian Academy of Sciences, Moscow, Russia; 2 Moscow Scientific and Clinical Antituberculosis Center, Department of Health of Moscow, Moscow, Russia; 3 Research Institute for Phthisiopulmonology, I. M. Sechenov Moscow Medical Academy, Moscow, Russia

**Keywords:** Biochips, Hybridisation, Extensively drug-resistant tuberculosis, Oligonucleotide microarrays, XDR-TB

## Abstract

**Background:**

The steady rise in the spread of multidrug-resistant tuberculosis (MDR-TB) and extensively drug-resistant tuberculosis (XDR-TB) requires rapid and reliable methods to identify resistant strains. The current molecular methods to detect MTB resistance to second-line drugs either do not cover an extended spectrum of mutations to be identified or are not easily implemented in clinical laboratories. A rapid molecular technique for the detection of resistance to second-line drugs in *M. tuberculosis* has been developed using hybridisation analysis on microarrays.

**Methods:**

The method allows the identification of mutations within the *gyrA* and *gyrB* genes responsible for fluoroquinolones resistance and mutations within the *rrs* gene and the *eis* promoter region associated with the resistance to injectable aminoglycosides and a cyclic peptide, capreomycin. The method was tested on 65 *M. tuberculosis* clinical isolates with different resistance spectra that were characterised by their resistance to ofloxacin, levofloxacin, moxifloxacin, kanamycin and capreomycin. Also, a total of 61 clinical specimens of various origin (e.g., sputum, bronchioalveolar lavage) were tested.

**Results:**

The sensitivity and specificity of the method in the detection of resistance to fluoroquinolones were 98% and 100%, respectively, 97% and 94% for kanamycin, and 100% and 94% for capreomycin. The analytical sensitivity of the method was approximately 300 genome copies per assay. The diagnostic sensitivity of the assay ranging from 67% to 100%, depending on the smear grade, and the method is preferable for analysis of smear-positive specimens.

**Conclusions:**

The combined use of the developed microarray test and the previously described microarray-based test for the detection of rifampin and isoniazid resistance allows the simultaneous identification of the causative agents of MDR and XDR and the detection of their resistance profiles in a single day.

## Background

Tuberculosis (TB) is a serious medical and public problem that threatens human health worldwide. Furthermore, the steady rise in the spread of multidrug-resistant tuberculosis (MDR-TB) and extensively drug-resistant tuberculosis (XDR-TB) requires rapid and reliable methods to identify resistant strains, as early identification allows adequate treatment, further preventing the transmission of the dangerous agent.

However, TB caused by resistant strains is difficult to treat, and the drug regimens are lengthy, toxic, and significantly expensive. Ideally, the prescription of second-line anti-TB drugs should be based on knowledge of the causative agent’s resistance and the patient’s treatment history. At present, the most potent drugs include fluoroquinolones (FQs) and injectable antibiotics, kanamycin (KAN) and capreomycin (CAP).

Approximately 80% of FQ-resistant strains bear mutations within the quinolone resistance-determining region (QRDR) of the *gyrA* gene [1], yet it has been increasingly reported that some FQ-resistant isolates bear mutations in the *gyrB* gene. Although the contribution of these mutations to resistance is under discussion [2,3], the role of five mutations (D500A, N538D, N538T, T539P, E540V) was confirmed by a DNA gyrase inhibition assay [4-7]. Thus, including the *gyrB* gene as a target appears reasonable to improve the molecular detection of FQ resistance [8].

The most commonly reported mutations that cause resistance to injectable aminoglycosides and CAP are a1401g, c1402t and g1484t in the *rrs* gene [9]. Less frequently, mutations within the *eis* promoter region and *tlyA* gene are also considered to be responsible for resistance [10,11]. It is expected that the sensitivity of KAN resistance detection will increase from 58% to 87% if the *eis* locus is included in the analysis [1]. Additionally, the mutations in the *tlyA* gene are only associated with CAP resistance [12]; these mutations are rare and are located throughout the entire gene, making their identification awkward.

The current molecular methods to detect MTB resistance either do not cover an extended spectrum of mutations to be identified [13] or are not easily implemented in clinical laboratories, mainly due to their cost (e.g., sequencing and pyrosequencing) [14,15].

This report describes a rapid microarray technique to detect the resistance to FQs and second-line injectable drugs (KAN and CAP) in MTB. This technique is the perfect complement to the TB-Biochip test for the analysis of rifampin/isoniazid resistance [16], which has been used in Russia for more than 5 years. The assay developed analyses mutations in the *gyrA* (codons 70-102) and *gyrB* (codons 485-543) genes responsible for FQ resistance and mutations in the *rrs* gene and the *eis* promoter locus that are associated with the resistance to aminoglycosides and CAP. Because of the accurate, unambiguous identification of a wide spectrum of relevant mutations, the uncomplicated interpretation of the results, and the high-throughput nature, reliability and reproducibility, this method can easily be implemented in any clinical laboratory that is familiar with PCR.

## Methods

### Bacterial isolates, clinical samples, DNA isolation and microscopy

For the present study, 65 *M. tuberculosis* clinical isolates were chosen. The isolates were obtained from sputum samples collected from previously treated patients at the Moscow Scientific and Clinical Antituberculosis Center and two specialised antituberculosis clinics in Moscow. *M. tuberculosis* strain H37Rv was used as a control for the microbiological and genetic tests. The stored isolates were subcultured on Lowenstein-Jensen solid medium and incubated at 37°C for 2 to 4 weeks.

A total of 61 clinical samples (sputum, biopsy, bronchioalveolar lavage, caseating material, urine and cavitary walls) were obtained from patients (primary and previously treated) attending the Research Institute for Phthisiopulmonology, Moscow between January and March 2012. These samples were used only for tests of analytical sensitivity.

The clinical samples were processed according to the international guidelines using the N-acetyl-L-cysteine-NaOH decontamination procedure (final NaOH concentration: 1%). The clinical specimens were divided into two groups: one used for smear preparation and the other for DNA isolation. The smear grading was performed using WHO recommendations [17].

DNA extraction from the analysed isolates and clinical samples was performed using the “Proba-NK” DNA extraction kit (DNA-Technology Company, Russia). The DNA concentrations were determined spectrophotometrically at 260 nm, and the DNA samples were stored at -20°C. All isolates were analyzed by spoligotyping [18].

According to the Ethics Committees of Department of Health of Moscow and I. M. Sechenov Moscow Medical Academy, this research does not require ethical approval. All samples used in this study were without any personal information about the patients, in particular without any ID by name, address, i.e. anonymous samples.

### Drug susceptibility testing (DST)

First-line DST for rifampicin(RMP), isoniazid(INH) and ethambutol(EMB) was performed using Bactec MGIT 960 according to the manufacturer’s instructions. Among the 65 isolates, 46 were multidrug-resistant, whereas 3 and 4 isolates were resistant to only INH and RMP, respectively; 12 isolates were sensitive (See Additional file 1: Table S1).

The resistance of the isolates to ofloxacin(OFX) was determined by the absolute concentration method, and the breakpoint concentrations were 2 and 10 mg/L.

Levofloxacin (LVX), moxifloxacin (MFX), KAN and CAP MICs for all the isolates were determined using the Bactec MGIT 960 automated system. For KAN and CAP, 2.5 mg/L was used as the breakpoint concentration [19]; for LVX and MFX, the concentrations were 2 mg/L and 0.25 mg/L, respectively [20].

### Oligonucleotide design

The melting temperatures were calculated, and the secondary structures of the designed oligonucleotides were estimated using an OligoAnalyzer (Integrated DNA Technologies, http://eu.idtdna.com/analyzer/Applications/OligoAnalyzer/). The lengths of the oligonucleotides were adjusted to maintain the difference of the melting temperatures within 2 to 3°C.

The oligonucleotides used for immobilisation on the biochip and the primers for amplification were synthesised and purified as described [21]. The molecular masses of the oligonucleotides were measured with a matrix-assisted laser desorption ionisation–time of flight (MALDI-TOF) mass spectrometer (Compact MALDI 4; Kratos Analytical, Chestnut Ridge, NY) using sinapinic acid or 2-amino-5-nitropyridine as the matrix.

### Biochip design and interpretation of hybridisation results

**Figure 1:**
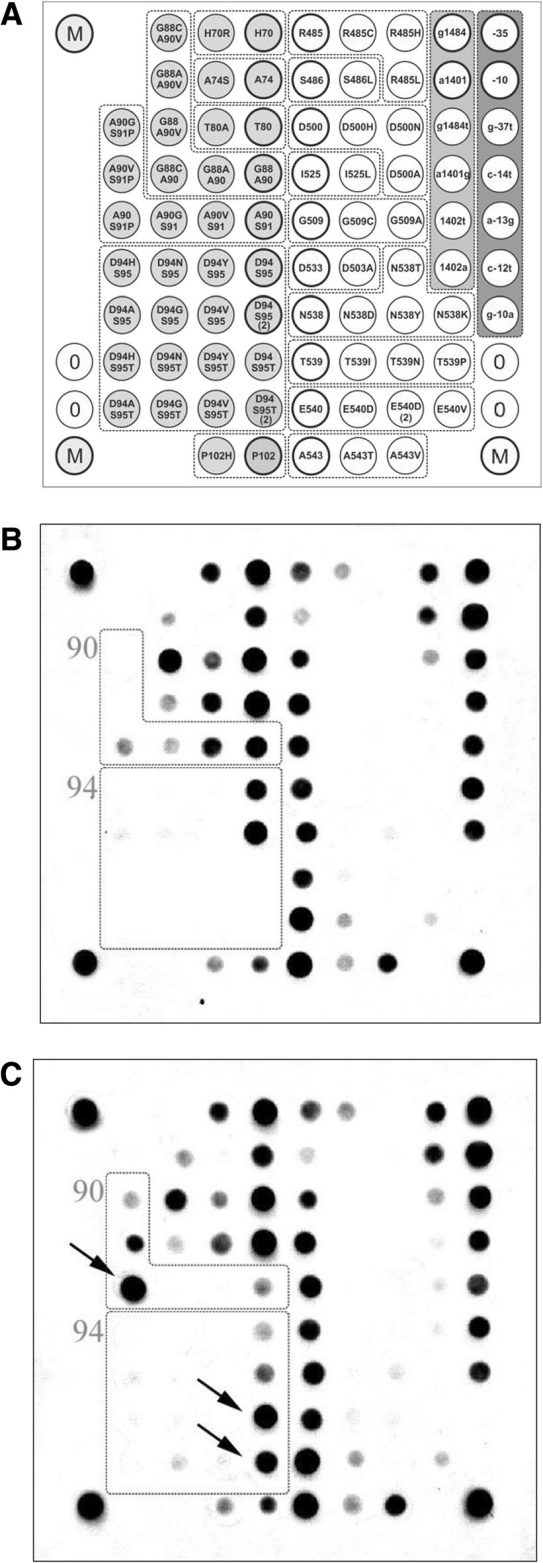
**Analysis of *Mycobacterium tuberculosis *drug susceptibility by hybridisation on a biochip.** (**A**) The scheme of the biochip for hybridisation. The subgroups are depicted as boxes. Each subgroup corresponds to a single variable amino acid position or variable nucleotide. One gel element within each subgroup contained an oligonucleotide matching the wild-type sequence (bold circles). (**B**) Hybridisation of a wild-type DNA sample on the biochip. (**C**) Hybridisation of a DNA sample containing the following mutations indicated by arrows: *gyrA* (S91P, TCG > CCG) and *gyrA* (S95T, AGC > ACC).

The biochip for the detection of mutations in *M. tuberculosis* leading to FQ and AMG resistance consisted of 83 gel pads with immobilised oligonucleotides (See Additional file 2: Table S2), 3 marker pads (M), and 2 reference gel pads without oligonucleotides for processing the hybridisation image (Figure 1). This biochip allows the detection of 16 and 23 mutations in the QRDR regions of the *gyrA* and *gyrB* genes, 4 mutations in the *rrs* gene and 5 mutations in the promoter region of the *eis* gene. The 4 clusters of analysed loci consisted of 36, 33, 6 and 7 array elements. The clusters can be subdivided into groups in which one element contains oligonucleotides with the wild-type sequence and the other pads contain sequences with mutations at the same position (2-5 bp). The interpretation of the fluorescence following the analysis was described previously [22].

### Biochip manufacture

The biochips were manufactured as described previously [21] and assembled with 30 μL hybridisation chambers (Biochip-IMB, LLC, Moscow, Russia). Each biochip contained semispherical gel elements of 150 μm in diameter placed 300 μm apart. Quality control was performed by measuring the fluorescence of reporter molecules on each gel element using the TestChip software provided by Biochip-IMB, LLC.

### PCR amplification

Multiplex asymmetrical PCR with universal adapters was used to amplify a 203 bp fragment of the *gyrA* gene, 313 bp of *gyrB*, 280 bp of *rrs* and 176 bp of *eis*. The primers used in the multiplex PCR are listed in Additional file 3: Table S3. The 30 μl reaction contained 15 μl Qiagen Multiplex Mastermix, 8 μM fluorescently labelled dUTP-ImD#49 (Biochip-IMB, LLC, Moscow), 100 μM *gyrA* and *rrs* primers, 50 μM *gyrB* and *eis* primers, 50 μM Uni-F and 2 mM of Uni-R primers, and 3 μl DNA sample. The cycling conditions were as follows: denaturation at 95°C for 5 min; 40 cycles of denaturation at 95°C for 30 s, annealing at 64°C for 30 s and extension at 72°C for 30 s; then 40 cycles of denaturation at 95°C for 30 s, annealing at 50°C for 30 s and extension at 72°C for 30 s; and a final extension step at 72°C for 5 min.

### Hybridisation on the biochip and registration of the results

The hybridisation mixtures were prepared by adding 10 μL of the PCR mixtures to 20 μL 1.5 M guanidine thiocyanate (GuSCN), 0.075 M HEPES (pH 7.5), and 7.5 mM EDTA. The biochip hybridisation chamber was filled with the mixture, and the assembly was incubated for 10 to 16 h at 37°C. The chamber was then removed, and the microarray surface was washed twice (approximately 30 s each) with water at 37°C and air-dried. The fluorescent pattern of the biochips was registered using a fluorescence analyser setup and specialised software “ImaGeWare” (Biochip-IMB, LLC).

### Sequencing

The fragments of the genes that determine resistance (*gyrA*, *gyrB*, *rrs*, and *eis*) were amplified using the corresponding primers (See Additional file 3: Table S3) and then subjected to dideoxy-sequencing using one of the terminal primers and the ABI PRISM® BigDye™ Terminator v. 3.1 (Applied Biosystems, Foster City, CA, USA) followed by analysis with the 3730 automatic DNA analyzer.

## Results

### Determination of MICs using Bactec MGIT 960

Among the 65 *M. tuberculosis* clinical isolates that were obtained from sputum samples, 42 (65%) were resistant to OFX, and most of the OFX-resistant strains were also resistant to LVX (n = 37) and MFX (n = 40), see Table 1. There was a strong correlation between the minimum inhibitory concentrations (MICs) of both fluoroquinolones. The MIC values for LVX and MFX were 4-16 mg/L and 0.5-4 mg/L, respectively, for many of the strains. One isolate was tolerant to 32 mg/L LVX and 8 mg/L MFX (the maximal MIC that was found).

**Table 1:** **Correlation of the detected mutations in the *gyrA *and *gyrB *genes with phenotypic DST**

**Mutation**		**No. of isolates**	**Resistance (mg/L)**	**MIC Bactec MGIT 960 (mg/L)**	
***gyrA***	***gyrB***		**OFX**	**LVX**	**MFX**
D94G	wt	1	10	**8**	**2**
D94G^a^	wt	12	2 to 10	**4** to **16**	**2**
D94A^a^	wt	6	2	2	**0.5** to **2**
D94N^a^	wt	1	2	**8**	**4**
D94H^a^	wt	1	10	**4**	**2**
D94Y^a^	wt	1	2	**4**	**1**
A90V^a^	wt	9	2 to 10	2 to **8**	**0.5** to **2**
S91P^a^	wt	1	2	2	**1**
G88C	wt	1	10	**8**	**4**
A90V + D94G^a^	wt	1	10	**>32**	**8**
A90V + D94Y^a^	wt	1	2	2	**1**
H70R + G88A^a^	wt	2	2	2	**1** to **2**
H70R + A90V^a^	wt	1	2	**8**	**2**
S95T	D500H	1	2	**4**	**0.5**
S95T	R485H	1	2	1	0.25
S95T	N538D	1	2	2	**0.5**
S95T	wt	1	2	1	0.125
S95T	wt	17	S	≤0.5	≤0.25
wt	wt	6	S	≤1	≤0.25
wt	wt	(H37Rv)	S	0.5	0.125

^a^additional mutation S95T.

Resistance to OFX was detected by the absolute concentration method. For LVX and MFX, resistance was defined as LVX (>2 mg/L) and MFX (>0.25 mg/L). The MICs considered to reflect resistance are highlighted in bold.

Resistance to KAN was detected in 34 (52%) isolates, with 18 strains demonstrating a high level of resistance to KAN (MIC >80 mg/L, see Table 2) and 17 also showing resistance to CAP (the MICs varied from 2.5 to 20 mg/L). The strains resistant to low and medium concentration of KAN (MIC: 2.5-10 mg/L) were also sensitive to CAP.

**Table 2:** **Correlation of detected mutations in the *rrs *and *eis *genes with phenotypic DST**

**Gene (mutation)**	**No. of isolates**	**MIC Bactec MGIT 960 (mg/L)**	
		**KAN**	**CAP**
*rrs* (a1401g)	17	**>80**	**3-20**
*eis* (g-10a)	10	**5 to >80**	0.6 to 2.5
*eis* (c-12t)	2	1.3 to **5**	1.3
*eis* (c-14t)	2	**20**	1.3
	1	0.63	1.3
*eis* (g-37t)	3	**10** to **20**	1.3 to 2.5
No mutation	29	0.3 to 2.5	0.6 to 2.5
	1	**5**	1.3
	(H37Rv)	2.5	2.5

The MICs considered to reflect resistance are highlighted in bold.

### Detection of mutations by biochip analysis

The mycobacterial target DNA loci responsible for the emergence of resistance to FQs, AMG and CAP were analysed by hybridisation on the developed microarray. The procedure consisted of two steps: (1) a multiplex PCR of the *gyrA*, *gyrB*, and *rrs* gene segments and the *eis* promoter locus and (2) hybridisation of fluorescently labelled single-stranded PCR products on the microarray.

The hybridisation pattern corresponding to *M. tuberculosis* H37Rv wild-type DNA is shown in Figure 1B. Within each group of gel elements, the strongest fluorescence signal corresponds to the gel pad containing a probe with the wild-type sequence. Hybridisation of a sample bearing a *gyrA* A90V mutation is shown in Figure 1C for comparison. The arrows point to pads on which the amplified DNA hybridised with oligonucleotide probes bearing mutant sequences. In group 90, the maximal signal corresponds to the oligonucleotide probe with a sequence leading to a A90V mutation. The analysis of 94 groups indicated that the DNA contains a S95T polymorphism.

### Analytical sensitivity

The performance of the biochip-based test was evaluated using 61 clinical samples characterised by smear microscopy; the results are shown in Table 3. The overall concordance with the smear microscopy for the biochip-based assay ranged from 100% with high AFB counts (3+) to 67% (1+). There were no differences in the sensitivity of the assay between the samples from different clinical material and collected from patients with newly detected TB and those previously treated for TB.

**Table 3:** Performance of the biochip-based assay depending on the concentration of acid-fast bacilli in clinical samples (n = 61)

**Smear grade**	**No. of samples**	**Positive result on biochip**	**Negative result on biochip**	**Sensitivity**
3+	19	19	0	100%
2+	13	10	3	77%
1+	12	8	4	67%
1-12 bacilli/300 fields	8	6	2	75%
negative	9	1	8	11%

The analytical sensitivity of this method was estimated by assaying 10-fold serial dilutions of the purified genomic DNA of *M. tuberculosis* strain H37Rv; four replicates were used for each dilution. The hybridisation results obtained with 300 genomic equivalents were unambiguous.

### Correlation of phenotypic DST with molecular assays

Data obtained from the biochip analysis and DST data for each strain are listed in Additional file 1: Table S1, and comparative summary is presented in Tables 1, 2 and 4.

**Table 4:** Performance of biochips in comparison with DST

**Biochip result**^**a**^	**Number of isolates with the indicated result**											
	**OFX**		**LVX**		**MFX**		**KAN**		**CAP**^**b**^		**XDR**	
	**R**	**S**	**R**	**S**	**R**	**S**	**R**	**S**	**R**	**S**	**R**	**S**
R	41	0	24	17	40	1	33	2	16	1	26	2
S	1	23	0	24	0	24	1	29	0	48	2	37
Sensitivity (%)	98		100		100		97		100		93	
Specificity (%)	100		59		98		94		94		95	

^a^ - R, isolates with mutations in *gyrA*, *gyrB* (FQ), *rrs*(KAN and CAP), *eis*(KAN); S, isolates without mutations.

^b^ - Isolates with mutations in *eis* were considered sensitive in biochip analysis.

*gyrA* mutations conferring FQ resistance (38 of 42 strains) were found in 90% of the samples. No mutations in the QRDR regions of the *gyrB* were identified both by the biochip and the sequencing of DNA fragments contained from 475 to 585 codons. The mutation frequencies correlate with the previously reported data [8]: D94G and A90V were most frequently found in 23 strains. All 12 strains with D94G were resistant to LVX (MIC: 4-16 μg/mL), in contrast to 6 strains with D94A, which were sensitive to LVX (MIC: 0.5-2 μg/mL). The S95T polymorphism, which does not lead to resistance, was detected in 17 of 23 sensitive to FQ strains.

A strain with a rare G88C mutation [23,24] was resistant to all three FQs, and the rare mutation H70R [25] was found in three strains only in combination with G88A or A90V. Two more strains with double mutations in *gyrA* carried A90V with D94 substituted with G or Y. A90V + D94G showed higher resistance levels compared to the strains with single A90V or D94G mutations. The MICs of the A90V + D94Y strain were close to those of the A90V strains.

Of 41 strains, 3 had mutations in the *gyrB* gene, with only an S95T substitution in *gyrA*. Sequencing for confirmation of the biochip data included a fragment from 59 to 132 codons of *gyrA*. All three strains were resistant to 2 μg/mL OFX. The strain with D500H was also resistant to LVX and MFX, which was shown in an earlier study [3]. Additionally, we found a strain bearing R485H that was resistant to OFX and sensitive to LVX and MFX. The mutation R485H was previously found in an OFX-resistant strain [14]. The third strain with the well-characterised N538D mutation was also resistant to MFX.

Therefore, mutations were detected in 41 of the 42 strains resistant to FQs. In 24 sensitive strains, no false-positive results were obtained.

Mutations were found in 33 of 34 KAN-resistant strains: an *rrs*(a1401g) mutation was found in 17, with mutation in the *eis* promoter region in 16 strains. The *eis*(g-10a) mutation was found in 10 strains. With regard to KAN, no mutations were found either by biochip analysis or sequencing in 30 sensitive and one resistant strain. The strains with *rrs*(a1401g) mutations possessed a high level of KAN resistance (MIC >80 μg/mL) and were also resistant to CAP, with MICs ranging from 3 to 20 μg/mL.

The strains with *eis* mutations were sensitive to CAP and resistant to KAN, with MICs varying over a wide range (0.63 to 20 μg/mL). Although strains with *eis*(g-10a) and (g-37t) mutations were KAN resistant (n = 10 and 3, respectively), we also found KAN-sensitive strains with the same mutations. The variation in the KAN MICs for strains with the same mutation could be an unknown effect of the genetic background.

## Discussion

A rapid molecular technique to detect resistance to second-line drugs in MTB using hybridisation analysis on microarrays was developed in this study. The method allows the identification of mutations within the *gyrA* and *gyrB* genes (the regions responsible for FQ resistance), and mutations within the *rrs* gene and the *eis* promoter region associated with the resistance to injectable aminoglycosides and a cyclic peptide, capreomycin.

An original primer set was designed to obtain all four specific amplicons using multiplex PCR. The resulting PCR products were fluorescently labelled, predominantly single stranded, and were used for the hybridisation analysis on the microarray.

The oligonucleotide probes immobilised on the microarray were designed to identify nucleotide substitutions in codons 70, 74, 80, 88, 90, 91, 94, 95 and 102 for the *gyrA* gene, in 485, 500, 509, 525, 533, 538, 539, 540, and 543 codons for the *gyrB* gene, at positions 1401, 1402, and 1484 from transcription start site for the *rrs* gene and within the -10 and -35 promoter regions of the *eis* gene. The developed set of primers is theoretically capable of providing a sensitivity of 96% in the detection of fluoroquinolone resistance [14] and 87% and 55% for KAN and CAP resistance, respectively [1].

The role of a number of the *gyrB* and *eis* mutations with regard to the resistance phenotype remains under discussion. Only five mutations in *gyrB* gene (D500A, N538D, N538T, T539P, E540V) were confirmed for FQ resistance by a DNA gyrase inhibition assay [4-7]. Nevertheless, we extended a spectrum of identified *gyrB* mutations since one cannot exclude possibility that disputable mutations could alter the resistance phenotype in combination with other mutations, as was reported for GyrA (A74S) in combination with S95T [3] and T80A with mutations in codon 90 of the *gyrA* gene [4,26,27]. A large number of identified mutations does not lead to the decrease of analytical sensitivity. An updated data concerning contribution of particular mutation to the resistance will demand only adjusting the threshold in software for determination of this mutation on a chip, without redesigning the microarray and PCR primer system.

The performance of the method in revealing resistance in MTB was examined using 65 clinical isolates that differed in their resistance spectra. 46 of them were MDR, 3 and 4 strains were monoresistant to RMP and INH, correspondingly. Beijing genotype was predominant among all strains with strong difference between MDR and non-MDR strains: 74% versus 50%.

26 of 28 XDR strains were correctly identified by biochip analysis (sensitivity - 93%). Two MDR strains resistant to only FQs were identified as XDR, both bearing mutations in *eis* gene. The specificity of XDR detection was 95%.

Among the FQ-resistant strains, mutations in *gyrA* codons 90, 91, and 94 were observed in 86% cases (36 of 43). Four isolates (9%) contained substitutions within codons 70 and 88 (the *gyrA* gene), and two isolates had mutations in the *gyrB* gene only (D500H, and N538D). One more strain bearing R485H substitution in *gyrB* gene was resistant to OFX and sensitive to LVX and MFX; however, the role of this mutation in FQ resistance was not confirmed by a gyrase assay [2,3]. Five strains with double mutations in *gyrA* were detected. They could be either true double mutant, either the result of heteropeaks due to the presence of different clones [8]. However, the spoligotyping revealed the unambiguous pattern for every strain. The sensitivity and specificity of the method relating to FQ resistance were 98 and 100%, respectively.

It was found that the a1401g mutation in the *rrs* gene correlates with the resistance to KAN and CAP in MTB, as was previously demonstrated [1,15].

Most of the strains bearing *eis* mutations were resistant to KAN (16 from 18) yet sensitive to CAP, but we also found two KAN-sensitive strains with *eis*(c-12t) (1 of 2 strains) and *eis*(c-14t) (1 of 3 strains) mutations. From the published data, *eis*(c-12t) is more often found in sensitive strains than in resistant strains [1,10,15,28], with only one report indicating that all 4 strains with this mutation were resistant to KAN [29]. Most of the strains with *eis*(c-14t) were KAN resistant [1,10,29], and only one of approximately ten strains were found to be sensitive in two reports [15,28]. In our study, *eis*(g-10a) was the second most frequent mutation found in the KAN-resistant strains (29%, n = 10), and all strains with this mutation were resistant, though the KAN MIC values varied widely. In previous studies, all the strains [10,29] or most of the strains [1] with this mutation were resistant to KAN, whereas Engstrom [15,28] reported that 85% of the strains were sensitive. We observed that the *eis*(g-37t) mutation was quite rare (9%, n = 3) but always was associated with resistance to KAN. Such correlation is in full accordance with the conclusions of other researchers [1,10,15,28,29]. Therefore, the involvement of mutations in the *eis* promoter region in the development of resistance to KAN remains unclear and requires additional statistically valid tests.

The sensitivity of the method concerning KAN resistance was high (96%), and the test was specific. The sensitivity for CAP was 100% because all 17 isolates with the *rrs*(a1401g) mutation tolerated CAP at concentrations of 3-10 μg/mL.

The analytical sensitivity of the method, or the minimal amount of bacterial genome equivalents that can be reliably identified by the technique, was 300 per assay. The obtained value allowed us to suppose that clinical samples could be analysed using the method in addition to isolates. To obtain experimental evidence, 61 clinical specimens of various origin (e.g., sputum, bronchioalveolar lavage) were tested. The diagnostic sensitivity of the assay ranged from 67 to 100% and depended on the number of mycobacterial cells used per analysis. This sensitivity depending on sputum grade was comparable to Genotype® MTBDRPlus kit [30]. However, the performance of the method in smear negative samples was found to be insufficient. So, to be used as a direct method, the analysis of smear-positive or culture-positive samples for drug resistance profiling could be preferable.

The developed approach fits easily in institutions that are already using biochip-based kits for *M. tuberculosis* DST [16]. Moreover, the assay can be applied to any laboratory utilizing molecular genetic techniques for analysis of tuberculosis causative agent, by addition of simple and inexpensive (less than 10 k US dollars) fluorescence analyzer equipped with specialized software. On the other part, the method requires fully equipped DNA manipulation/PCR laboratory and trained staff to perform the test.

## Conclusions

In conclusion, we developed a sensitive and specific microarray-based technique for the identification of resistance to FQ and the second-line injectable aminoglycosides KAN and CAP in *Mycobacterium tuberculosis*. The knowledge of the MTB resistance spectrum is important for the effective treatment of MDR- and XDR-TB. The developed microarray is considered to be the perfect complement for the commercially available diagnostic system TB-Biochip (MDR) [22]. The combined use of the developed microarray test and the TB-Biochip (MDR) allows the simultaneous identification of the MDR and XDR causative agents and the detection of their resistance profiles in a single day.

## Competing interests

The authors declare that they have no competing interests.

## Authors’ contributions

ZDV was the primary researcher, conceived the study, designed the assay, performed laboratory experiments, conducted data analysis and drafted the manuscript for publication. AOV performed evaluation of the assay. KAV and PSA participated in sample collection. IYD & KLY performed the DST experiments. ZAS and MVM reviewed the initial and final drafts of the manuscript. GDA participated in the design and oversaw the execution of the study, and helped to draft the manuscript. All authors read and approved the final manuscript.

## Pre-publication history

The pre-publication history for this paper can be accessed here:

http://www.biomedcentral.com/1471-2334/13/240/prepub

## Supplementary Material

Additional file 1: Table S1 Clinical isolates used for evaluation of the biochip. Table in PDF format containing list of all isolates used in this study with their resistance profiles and biochip analysis data. Click here for file

Additional file 2: Table S2 Oligonucleotides used for the microarray. Table in PDF format containing list of oligonucleotide probes immobilized in biochip pads for detection of mutations in *gyrA*, *gyrB*, *rrs* and *eis* genomic *loci*. Click here for file

Additional file 3: Table S3 Primers used for the amplification of the *gyrA*, *gyrB*, *rrs* and *eis* fragments. Table in PDF format containing list of primers used in multiplex PCR system and primers used for sequencing of DNA fragments of *gyrA*, *gyrB*, *rrs* and *eis* genomic loci. Click here for file
