# Supplementary material for: Detection of second-line drug resistance in Mycobacterium tuberculosis using oligonucleotide microarrays
Source: BMC Infect Dis. 2013 May 24;13:240. doi: 10.1186/1471-2334-13-240 (PMC3671172; doi:10.1186/1471-2334-13-240)
Supplement: Additional file 1: Table S1 — Clinical isolates used for evaluation of the biochip. Table in PDF format containing list of all isolates used in this study with their resistance profiles and biochip analysis data. [file 1471-2334-13-240-S1.pdf]

Table S1 - Clinical isolates used for evaluation of the biochip.

	No.	Spoligotyping[a]		Resistance[b]				MIC Bactec MGIT 960 (mg/L)[b]				Mutations detected by biochip			
		SIT	Lineage	INH	RMP	EMB	OFX mg/L	LVX	MFX	KAN	CAP	gyrA	gyrB	rrs	eis
XDR	2305	65	T1	R	R	R	2	2	1	10	2,5	H70R+G88A[c]	-	-	g-10a
	2306	1	Beijing	R	R	R	2	2	1	5	1,25	D94A[c]	-	-	c-12t
	2308	878	X1	R	R	R	2	4	0,5	>80	10	[c]	D500H	a1401g	
	2315	1	Beijing	R	R	R	2	4	1	>80	10	D94Y[c]	-	a1401g	
	2319	1	Beijing	R	R	R	2	2	0,5	>80	5	A90V[c]	-	a1401g	
	2327	1	Beijing	R	R	R	10	8	2	>80	5	A90V[c]	-	a1401g	
	2328	1	Beijing	R	R	R	2	2	1	>80	5	A90V+D94Y[c]	-	a1401g	
	2331	1	Beijing	R	R	R	10	4	2	10	1,25	D94H[c]	-	-	g-37t
	2332	65	T1	R	R	R	2	2	2	10	1,25	H70R+G88A[c]	-	-	g-10a
	2333	1	Beijing	R	R	R	10	>32	8	>80	5	A90V+D94G[c]	-	a1401g	
	2338	1	Beijing	R	R	R	2	2	0,5	5	1,25	D94A[c]	-	-	
	2339	1	Beijing	R	R	R	2	4	2	>80	10	A90V[c]	-	a1401g	
	2347	1	Beijing	R	R	R	2	1	0,25	>80	10	[c]	-	a1401g	
	2349	252	LAM9	R	R	R	10	16	2	>80	10	D94G[c]	-	a1401g	
	2352	891	LAM9	R	R	R	10	8	2	>80	10	D94G[c]	-	a1401g	
	2354	1	Beijing	R	R	R	2	4	2	>80	10	D94G[c]	-	a1401g	
	2358	65	T1	R	R	R	2	2	0,5	>80	20	D94A[c]	-	a1401g	
	2360	237	U (likely H3)	R	R	R	2	2	1	20	1,25	D94A[c]	-	-	c-14t
	2365	1	Beijing	R	R	R	10	4	1	10	1,25	A90V[c]	-	-	g-10a
	2367	1	Beijing	R	R	R	10	4	2	>80	3	D94G[c]	-	a1401g	
	2368	1	Beijing	R	R	R	10	2	0,5	>80	2,5	A90V[c]	-	-	g-10a
	2369	1	Beijing	R	R	R	2	4	2	10	1,25	D94G[c]	-	-	g-10a
	2317	1	Beijing	R	R	S	2	8	2	>80	10	H70R+A90V[c]	-	a1401g	
	2318	1	Beijing	R	R	S	2	2	0,5	20	1,25	A90V[c]	-	-	c-14t
	2321	254	T5_RUS1	R	R	S	2	1	0,25	10	2,5	[c]	R485H	-	g-10a
	2342	1	Beijing	R	R	S	10	8	2	>80	5	D94G[c]	-	a1401g	
	2344	190	Beijing	R	R	S	10	4	2	10	1,25	D94G[c]	-	-	g-37t
	2361	1	Beijing	R	R	S	10	4	2	>80	5	D94G[c]	-	a1401g	
MDR	2311	1	Beijing	R	R	S	2	8	4	1,25	1,25	D94N[c]	-	-	
	2341	1	Beijing	R	R	R	10	4	2	0,63	1,25	D94G[c]	-	-	c-14t
	2345	509	LAM9	R	R	S	2	4	0,5	2,5	1,25	A90V[c]	-	-	

	2355	237	U (likely H3)	R	R	R	10	16	2	2,5	1,25	D94G[c]	-	-	
	2357	1	Beijing	R	R	S	10	8	4	1,25	1,25	G88C[c]	-	-	
	2359	1	Beijing	R	R	R	10	4	2	1,25	1,25	D94G[c]	-	-	
	2314	265	Beijing	R	R	R	2	2	1	1,25	1,25	S91P[c]	-	-	
	2329	265	Beijing	R	R	R	2	2	2	1,25	1,25	D94A[c]	-	-	
	2334	265	Beijing	R	R	R	2	2	0,5	0,31	1,25	[c]	N538D	-	
	2343	1	Beijing	R	R	R	2	2	0,5	0,63	1,25	A90V[c]	-	-	
	2363	1	Beijing	R	R	R	2	2	1	1,25	1,25	A90V[c]	-	-	c-12t
	2326	35	H4	R	R	S	0	0,25	0,13	10	1,25	[c]	-	-	g-10a
	2336	1	Beijing	R	R	R	0	0,25	0,13	5	2,5	[c]	-	-	g-10a
	2351	1	Beijing	R	R	S	0	0,13	0,13	10	1,3	[c]	-	-	g-10a
	2309	265	Beijing	R	R	R	0	0,25	0,13	1,25	1,25	[c]	-	-	
	2320	1	Beijing	R	R	S	0	0,25	0,13	0,63	0,63	[c]	-	-	
	2335	1	Beijing	R	R	R	0	0,25	0,13	0,63	0,63	[c]	-	-	
	2350	467	H3	R	R	S	0	0,25	0,25	0,63	1,25	-	-	-	
non-MDR	2348	1	Beijing	R	S	S	2	4	2	>80	5	D94G[c]	-	a1401g	
	2346	1	Beijing	R	S	S	2	2	1	1,25	1,25	D94A[c]	-	-	
	2313	1	Beijing	R	S	S	0	0,25	0,25	10	0,63	[c]	-	-	g-10a
	2316	65	T1	R	S	S	0	0,25	0,13	1,25	1,25	[c]	-	-	
	2353	50	H3	S	R	S	0	0,5	0,25	1,25	1,25	-	-	-	
	2356	172	U	S	R	S	0	0,25	0,25	1,25	1,25	-	-	-	
	2366	254	T5_RUS1	S	R	S	0	0,25	0,13	1,25	1,25	[c]	-	-	
	2310	280	T1_RUS2	S	S	S	10	8	2	20	2,5	D94G	-	-	g-37t
	2307	1	Beijing	S	S	S	0	0,25	0,13	0,63	1,25	[c]	-	-	
	2312	280	T1_RUS2	S	S	S	0	0,5	0,13	1,25	1,25	[c]	-	-	
	2322	262	H4	S	S	S	0	0,25	0,13	1,25	1,25	[c]	-	-	
	2323	762	H4	S	S	S	0	0,25	0,13	1,25	1,25	[c]	-	-	
	2324	1	Beijing	S	S	S	0	0,25	0,13	0,63	0,31	[c]	-	-	
	2325	1	Beijing	S	S	S	0	0,25	0,13	1,25	1,25	[c]	-	-	
	2330	1	Beijing	S	S	S	0	0,25	0,13	0,63	0,31	-	-	-	
	2337	1	Beijing	S	S	S	0	0,25	0,13	1,25	2,5	[c]	-	-	
	2340	1	Beijing	S	S	S	0	0,5	0,06	1,25	1,25	[c]	-	-	
	2362	53	T1	S	S	S	0	0,5	0,13	1,25	1,25	-	-	-	
	2364	52	T2	S	S	S	0	1	0,13	1,25	1,25	-	-	-	

[a] Spoligoprofiles were assigned to phylogenetic families using SITVITWEB (http://www.pasteur-guadeloupe.fr:8081/SITVIT_ONLINE)
[b] Resistance obtained by absolute concentration method and MICs considered to reflect resistance are marked with grey colour.
[c] Additional mutation in *gyrA* - S95T.
