# Supplementary material for: Detection of second-line drug resistance in Mycobacterium tuberculosis using oligonucleotide microarrays
Source: BMC Infect Dis. 2013 May 24;13:240. doi: 10.1186/1471-2334-13-240 (PMC3671172; doi:10.1186/1471-2334-13-240)
Supplement: Additional file 2: Table S2 — Oligonucleotides used for the microarray. Table in PDF format containing list of oligonucleotide probes immobilized in biochip pads for detection of mutations in gyrA, gyrB, rrs and eis genomic loci. [file 1471-2334-13-240-S2.pdf]

Table S2 - Oligonucleotides used for the microarray.

Oligonucleotide	Amino acid ⁄ nucleotide position	Amino acid substitution	Nucleotide substitution	Sequence 5' to 3'	Length
gyrA gene					
H70	70	His(wt)		gaccgcagccacgccaagtcg	21
H70R	70	His - Arg	cac - cgc	accgcagccgcgccaagtc	19
A74	74	Ala(wt)		ccaagtcggcccggtcgg	18
A74S	74	Ala - Ser	gcc - tcc	gccaagtcgtcccggtcgg	19
T80	80	Thr(wt)		tgccgagaccatgggc	16
T80A	80	Thr - Ala	acc - gcc	gccgaggccatgggc	15
G88	88	Gly(wt)		cccgcacggcgacgcg	16
G88C	88	Gly - Cys	ggc - tgc	cccgcactgcgacgcg	16
G88A	88	Gly - Ala	ggc - gcc	cccgcacgccgacgcg	16
G88A A90V	88	Gly(wt)		cccgcacgccgacgtg	16
G88 A90V	88	Gly - Cys	ggc - tgc	cccgcacggcgacgtg	16
G88C A90V	88	Gly - Ala	ggc - gcc	cccgcactgcgacgtg	16
A90 S91	90	Ala(wt)		cgacgcgtcgatctacga	18
A90V S91	90	Ala -Val	gcg - gtg	cgacgtgtcgatctacga	18
A90G S91	90	Ala - Gly	gcg - ggg	cgacgggtcgatctacga	18
A90 S91P	90	Ala(wt)[a]		cgacgcgccgatctacg	17
A90V S91P	90	Ala -Val[a]	gcg - gtg	cgacgtgccgatctacga	18
A90G S91P	90	Ala - Gly[a]	gcg - ggg	cgacgggccgatctacg	17
D94 S95	94	Asp(wt)		cgatctacgacagcctg	17
D94H S95	94	Asp - His	gac - cac	cgatctaccacagcctg	17
D94A S95	94	Asp - Ala	gac - gcc	gatctacgccagcctg	16
D94N S95	94	Asp - Asn	gac - aac	tcgatctacaacagcctg	18
D94G S95	94	Asp - Gly	gac - ggc	gatctacggcagcctg	16
D94Y S95	94	Asp - Tyr	gac - tac	tcgatctactacagcctg	18
D94V	94	Asp -Val	gac - gtc	cgatctacgtcagcctg	17
D94 S95T	94	Asp(wt)		tctacgacaccctggt	16
D94H S95T	94	Asp - His[b]	gac - cac	cgatctaccacaccctg	17
D94A S95T	94	Asp - Ala[b]	gac - gcc	tctacgccaccctggt	16
D94N S95T	94	Asp - Asn[b]	gac - aac	tcgatctacaacaccctg	18
D94G S95T	94	Asp - Gly[b]	gac - ggc	gatctacggcaccctg	16
D94Y S95T	94	Asp - Tyr[b]	gac - tac	cgatctactacaccctg	17
D94V S95T	94	Asp -Val[b]	gac - gtc	cgatctacgtcaccctg	17
D94 S95(2)	95	Ser(wt)		tacgacagcctggtgcg	17
D94 S95T(2)	95	Ser - Thr	agc -acc	tacgacaccctggtgcg	17
P102	102	Pro		gcccagccctggtcgct	17

P102H	102	Pro - His	ccc - cac	ggcccagcactggtcgc	17
gyrB gene					
R485	485	Arg(wt)		gccgattgccgttccacg	18
R485C	485	Arg - Cys	cgt - tgt	gccgattgctgttccacg	18
R485H	485	Arg - His	cgt -cat	ccgattgccattccacg	17
R485L	485	Arg - Leu	cgt -ctt	gccgattgcctttccacg	18
S486	486	Ser(wt)		cgattgcagttccacggatccgc	23
S486F	486	Ser-Phe	tcc-ttc	cgattgcagtttcacggatccgcg	24
D500	500	Asp(wt)		gtagaaggtgactcggccg	19
D500H	500	Asp - His	gac - cac	gtagaaggtcactcggccg	19
D500N	500	Asp - Asn	gac - aac	cgtagaaggtaactcggccg	20
D500A	500	Asp - Ala	gac - gcc	tagaaggtgcctcggccg	18
G509	509	Gly(wt)		gcaaaaagcggtcgcgattc	20
G509C	509	Gly - Cys	ggt - tgt	tgcaaaaagctgtcgcgattc	21
G509A	509	Gly - Ala	ggt - gct	gcaaaaagcgctcgcgattc	20
I525	525	Ile(wt)		cggcaagatcatcaatgtgga	21
I525L	525	Ile - Leu	atc - ctc	cggcaagatcctcaatgtgg	20
D533	533	Asp(wt)		gcgcatcgaccgggtg	16
D533A	533	Asp - Ala	gac - gcc	cgcatcgcccgggtg	15
N538	538	Asn(wt)		gtgctaaagaacaccgaa	18
N538D	538	Asn - Asp	aac - gac	tgctaaaggacaccgaa	17
N538Y	538	Asn -Tyr	aac - tac	gtgctaaagtacaccgaag	19
N538K	538	Asn -Lys	aac - aaa	gtgctaaagaaaaccgaa	18
N538T	538	Asn - Thr	aac - acc	tgctaaagaccaccgaa	17
T539	539	Thr(wt)		ctaaagaacaccgaagttc	19
T539I	539	Thr - Ile	acc - atc	ctaaagaacatcgaagttca	20
T539N	539	Thr - Asn	acc - aac	gctaaagaacaacgaagtt	19
T539P	539	Thr - Pro	acc - ccc	ctaaagaaccccgaagtt	18
E540	540	Glu(wt)		aacaccgaagttcaggc	17
E540D	540	Glu - Asp	gaa - gat	aacaccgatgttcaggc	17
E540D(2)	540	Glu - Asp	gaa - gac	aacaccgacgttcagg	16
E540V	540	Glu - Val	gaa - gta	aacaccgtagttcaggc	17
A543	543	Ala(wt)		agttcaggcgatcatca	17
A543T	543	Ala - Thr	gcg - acg	aagttcagacgatcatca	18
A543V	543	Ala - Val	gcg - gtg	agttcaggtgatcatca	17
rrs gene					
1401	1401	-		cgcccgtcacgtcatgaa	18
a1401g	1401	-	cac - cgc	gcccgtcgcgtcatgaa	17
c1402t	1402	-	cac - cat	cgcccgtcatgtcatgaaa	19
c1402a	1402	-	cac - caa	cgcccgtcaagtcatgaaa	19
1484	1484	-		gattgggacgaagtcgtaaca	21

g1484t	1484	-	cgc - ctc	gattgggactaagtcgtaaca	21
eis gene					
10wt	-10 [c]	-		gcatatgccacagtcggatt	20
c-14t	-14	-	cca - cta	ggcatatgctacagtcggatt	21
a-13g	-13	-	cac - cgc	catatgccgcagtcggat	18
c-12t	-12	-	aca - ata	gcatatgccatagtcggattc	21
g-10a	-10	-	agt - aat	catatgccacaatcggattctg	22
35wt	-35	-		cgtaatattcacgtgcacgtagc	23
g-37t	-37	-	cgt - ctt	taatattcacttgcacgtagcc	22

[a] additional mutation Ser91Pro

[b] additional mutation Ser95Pro

[c] position relative the start codon
