# Supplementary material for: Detection of second-line drug resistance in Mycobacterium tuberculosis using oligonucleotide microarrays
Source: BMC Infect Dis. 2013 May 24;13:240. doi: 10.1186/1471-2334-13-240 (PMC3671172; doi:10.1186/1471-2334-13-240)
Supplement: Additional file 3: Table S3 — Primers used for the amplification of the gyrA, gyrB, rrs and eis fragments. Table in PDF format containing list of primers used in multiplex PCR system and primers used for sequencing of DNA fragments of gyrA, gyrB, rrs and eis genomic loci. [file 1471-2334-13-240-S3.pdf]

Table S3 - Primers used for the amplification of the *gyrA, gyrB*, *rrs* and *eis* fragments.

Primer	Sequence 5' to 3'	Length
gyrA-F	ctctgcgagcataatg-ggaggtgcgcgacgggctcaagc	39
gyrA-R	ggctgtacgctgtc-ccagcgggtagcgcagcgacca	36
gyrB-F	ctctgcgagcataatg-caaggctgtgtcctcggcgcaagcc	41
gyrB-R	ggctgtacgctgtc-cggtgcccagcgccgtgatgat	36
rrs-F	ctctgcgagcataatg-tctcagttcggatcggggtctgcaactcg	45
rrs-R	ggctgtacgctgtc-ccagttggggcgttttcgtggtgctcc	41
eis-F	ctctgcgagcataatg-gcgaaattcgtcgctgattctcgcagtggc	46
eis-R	ggctgtacgctgtc-cccggccagtcgtcctcggtcg	36
uni-F	ctctgcgagcataatg	16
uni-R	ggctgtacgctgtc	14
gyrA-F-seq	gtgcgcgacgggctcaagc	19
gyrA-R-seq	catcgccaacggggtcagcc	20
gyrB-F-seq	caaggctgtgtcctcggcgca	21
gyrB-R-seq	cacttgagtttgtacagcggcggttg	26
rrs-F-seq	tctcagttcggatcggggtctg	22
rrs-R-seq	gctctcgcccactacagacaagaa	24
eis-F-seq	gaaattcgtcgctgattctcgcagtggc	28
eis-R-seq	gccgcggccagtaggaacatc	21

The universal parts of primers are separated by hyphens
